# Programmable hierarchical plasmonic–photonic arrays *via* laser-induced film dewetting

**DOI:** 10.1515/nanoph-2022-0272

**Published:** 2022-07-15

**Authors:** Zeyu Zheng, Yu Miao, Jiyuan Yao, Jiamei Chen, Jialin Wen, Xiaodan Chen, Yanxin Lu, Xiaofang Jiang, Lingling Shui

**Affiliations:** Guangdong Provincial Key Laboratory of Nanophotonic Functional Materials and Devices, School of Information and Optoelectronic Science and Engineering, South China Normal University, Guangzhou 510006, P. R. China; Laboratory of Quantum Engineering and Quantum Material, School of Physics and Telecommunication Engineering, South China Normal University, Guangzhou 510006, China

**Keywords:** display, hierarchical nanostructure, information storage, laser-induced dewetting, photonic crystal, self-assembly

## Abstract

Hierarchical and periodic nanostructures of dielectrics or metals are highly demanded for wide applications in optical, electrical, biological, and quantum devices. In this work, programmable plasmonic–photonic hierarchical nanostructures are fabricated using a facile and effective method with high controllability and stable reproducibility. The fabrication involves colloidal self-assembly, metal film deposition, and pulsed laser-induced dewetting in sequence for controllably pairing metal nanostructures on dielectric nanospheres in either large area or a local precision. Au nanostructures including Au nanocrown (AuNC), large Au nanosphere (AuNS), and multiple small Au nanoparticles (AuNPs) have been paired one-on-one on assembled SiO_2_ nanosphere (SiO_2_NS) arrays, with size and shape controlled by correlating the laser fluence and irradiation time, and the Au film thickness. The fabricated hierarchical nanostructures demonstrate synergistic effect of the photonic effects from the monolayer SiO_2_NS arrays and the surface plasma resonance effect from the Au nanostructures. The dewetting induced metal film reshaping has been modeled theoretically corresponding to observed experimental results. We can directly “write” the plasmonic Au nanostructures on the photonic crystal array using a focused laser beam to form encode patterns, showing angle-dependent structural colors for anti-counterfeiting information storage and display in rigid/flexible and opaque/transparent devices. It provides a promising path to actively construct on-demand pixelated plasmonic–photonic arrays for optical multiplexing technology in sensing, information encryption, and display.

## 1 Introduction

Hierarchical periodic nanostructures with multilevel micro/nanoscale patterns have attracted intensive interest due to their unique pattern-dependent properties and their potential applications in photonics [1, 2], electronics [3, 4], biology [5, 6], and catalysis [7]. In particular, 2D planar photonic crystal (2D PC) is a highly ordered monolayer structure formed by periodic arrangement of media with different refractive index, giving rise to the photonic band gap [8] (PBG) in which light propagation is forbidden through the inner plane of PC and the strong electromagnetic dipole resonance (EMDR) as dielectric metasurface [9]. These photonic properties of PBG and EMDR provide the unique capability of manipulating electromagnetic waves at the nanometer scale. When PBG or/and EMDR locate at the visible region, 2D PC exhibit bright iridescence called structural color [10, 11]. Structural color exhibits unique characteristics of angle dependence, anti-photobleaching, and high color purity and shows fascinating applications in anti-counterfeiting and color display. Noble metal nanoparticles display surface plasma resonance (SPR) effect, which arises from the collective oscillation of conduction band electrons, providing an exciting platform for manipulating and amplifying light–matter interactions at the nanoscales [12, 13]. The frequency and intensity of SPR are strongly related to the particle shape, size, optical environment as well as the interparticle plasmon coupling [14, 15]. Compared to 2D PC and plasmonic nanoparticles, hierarchical periodic nanostructures incorporated well-assembled noble metal nanoparticles with 2D PC was expected to exhibit the strong plasmonic–photonic coupling effects, showing more advanced potentials in photonics [16], catalysts [17, 18], and sensors [11, 19]. Therefore, it is highly desirable to develop an effective approach to precisely program the spatial location and morphology of metallic nanoparticles in these hierarchical structures with high controllability and stable reproducibility.

Many efforts have been made to construct the hierarchical periodic plasmonic–photonic nanostructures in a facile and economic way with adjustable structural parameters. Compared to the top-down strategies, the self-assembly of nanoscale functional building blocks (e.g., dielectric particles [20], noble metal nanoparticles [21], and magnetic particles [22]) are attractive due to their quick, cheap, and large-area fabrication [23]. In 2020, our group reported a multilevel spherical photonic crystal structure anchored with gold nanoparticles for tailoring optical resonances by thermal-induced dewetting of metal films [16, 24]. In 2021, Utsav et al. reported a thermal crowning mechanism and achieved gold nanocrowns on the 2D periodic arrays of SiO_2_ nanospheres [25]. Thermal dewetting method allows for large-area fabrication but is not suitable for designable nanostructure morphology and micro-patterning. The long-time preparation process and poor structural controllability also limits its applications.

Compared to thermal dewetting, pulsed laser-induced dewetting is an effective patterning method for metallic structure fabrication owing to its fast and controllable process, large-area fabrication, focused area patternability, and low environmental dependence as well as easy operation [26, 27]. Laser-induced dewetting offers a multitude of possibilities to directly “write” different nanostructures in either time or special sequence by adjusting the processing parameters. In 2005, Henley S J et al. fabricated various metal nanoparticles by using pulsed laser-induced dewetting of thin metal films (Au, Ag, Ni, Mo) on plane substrates [28]. In 2013, Yong-Jun Oh et al. produced a well-ordered Co-Pt metal nanoparticles array with relatively uniform size on interference lithography templated substrate by pulsed laser-induced dewetting [29]. They found templated dewetting with more beneficial for producing a more uniform size and shape distribution compared to untemplated dewetting by laser. Up to now, most of the template fabrication methods are based on photolithography or electron beam lithography which suffer from the high cost, time-consuming, and strict equipment requirement. In this regard, the laser-induced patterning dewetting employing with self-assembly periodic templates is much promising in the fabrication of hierarchical and periodic structures with low-cost, timesaving, and large-area. Till now, the detailed investigation on the programmable reshaping mechanism as well as the optical properties of plasmonic nanostructures on photonic array by laser-induced dewetting is still missing.

Herein, we report a facile and effective method to fabricate hierarchical one-on-one pairing plasmonic–photonic nanostructures with high controllability and stable reproducibility *via* self-assembly produced photonic crystal structure and dewetting generated plasmonic Au nano-units. High quality monolayer SiO_2_NS array is prepared using a wetting-assisted interfacial self-assembly process, which provides evenly distributed curved nanosurfaces as supporting units for the formation of Au films. The plasmonic structures is prepared through ns-laser induced Au film dewetting, achieving various Au nanostructures of nanocrown, large nanosphere, and small nanoparticles defined by altering the laser fluence and irradiation time correlated with Au film thickness. The formation mechanism and the optical properties of these hierarchical nanostructures are investigated in both theoretical and experimental methods. Due to the strong plasmonic–photonic coupling, these hierarchical nanostructures possess diverse angle-dependent structural colors relative to both photonic and plasmonic structures. Various plasmonic structures can be encoded on the photonic surface with designed patterns, showing different colors and color contrast depending on viewing angles. These features allow for advanced anti-counterfeiting applications. Moreover, the hierarchical structures can be transferred to flexible and transparent *via* a simple pasting and peeling process. These spatially and timely programable hierarchical nanostructures with pixelated arrays offer remarkable potentials optical multiplexing technology.

## 2 Experimental

### 2.1 Preparation of self-assembly monolayer SiO_2_NS array

Monodisperse SiO_2_NSs with a diameter of ∼500 nm (Nanorainbow, *C* = 10%) was dispersed in absolute ethyl alcohol (EtOH, ≥99.7%, v/v) with solid content of 2.5–3.0 wt%. A (100) silicon wafer (Si) was cut into 2 mm × 2 mm square pieces for further experiments. The silicon substrate was cleaned by soaking in piranha solution (H_2_O_2_:H_2_SO_4_ = 3:7, v/v), then blow dried using nitrogen gun; and then treated by oxygen plasma (Harrick Plasma, PDC-002) for 8 min. The nanospheres self-assembly experiment was performed at an ambient temperature of 20 ± 2 °C and humidity of 50–60%. The silicon substrate with assembled monolayer SiO_2_NS array was heated in a tubular furnace (OTF-1200X, HF-Kejing) at 800 °C for 2 h, and sequentially cooled gently to remove the residual water on the substrate and enhance the physical properties.

### 2.2 Au film preparation

Thermal evaporation equipment (Electron beam section and Resistance Section composite coating system, Shen Yang Ju Zhi) was used to deposit a thin Au film on the upper surface of monolayer SiO_2_NSs at the vacuum condition and deposition rate of 0.02 nm/s in the deposition chamber. The thickness of the thin Au film can be controlled by the deposition time.

### 2.3 Laser-induced dewetting processing

The dewetting of the Au film on monolayer SiO_2_NSs was performed by using a Q-switched 532 nm ns laser (ns-laser) with a pulse duration of ∼10 ns and a repetition rate of 3 kHz (AO-W-532, CNI). The laser beam was focused onto the film by a convex lens (focal length *f* = 50.8 mm). A sample was placed on a computer-controlled XY displacement platform (PMC100-3, TRZH) to precisely control the dewetting region. An electronic shutter device (GCI-73 M, Heng Yang) was utilized to accurately control the irradiation time.

### 2.4 Characterization methods

The thickness and morphology of Au films obtained by thermal evaporation were analyzed using an atomic force microscopy (AFM, Multimode 8, Bruker). A scanning electron microscopy (SEM, Gemini 500, Carl Zeiss) was used to characterize the nanostructures. The angle-dependent spectra were measured using an angular resolution spectrometer (Angular Resolution Spectrometer R1, Ideaoptics). The polished clean silicon wafer was used as a reference. The photographs of the structural colors of the constructed hierarchical nanostructures were taken using a smartphone (Huawei, P20 Pro) under different viewing angles.

## 3 Results and discussion

**Figure 1:**
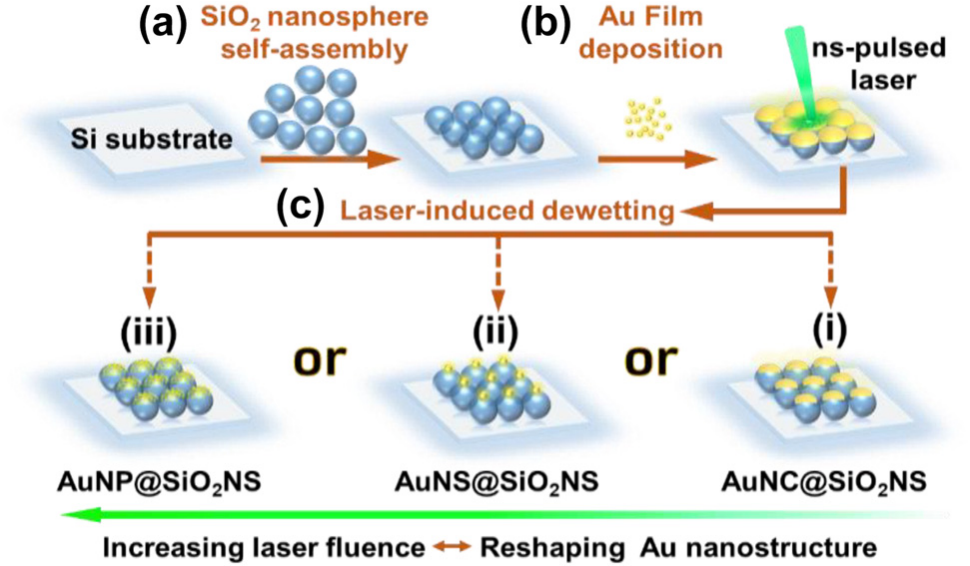
Schematic diagram of the hierarchical plasmonic–photonic nanostructure arrays fabrication process. (a) Wetting-assisted self-assembly of SiO_2_NSs to prepare 2D photonic crystal array. (b) Au film deposition on SiO_2_NSs by thermal evaporating (Au@SiO_2_NS). (c) Au film dewetting induced by ns-pulsed laser irradiation to obtain various hierarchical nanostructures of (i) AuNC@SiO_2_NS, (ii) AuNS@SiO_2_NS and (iii) AuNPs@SiO_2_NS with Au film reshaping tuned by the laser fluence.

Figure 1 illustrates the schematic fabrication process of the well-ordered hierarchical plasmonic–photonic nanostructure arrays. Monolayer 2D photonic crystal structure was prepared *via* a wetting-assisted self-assembly of SiO_2_NSs at gas–liquid interface [30]. Afterwards, Au was deposited using a thermal evaporation technique to form evenly distributed nanofilm on each SiO_2_NS. The dewetting of Au film was then achieved by nanosecond pulsed (ns-pulsed) laser irradiation. By controlling the Au film thickness and the laser-induced dewetting parameters (laser fluence and irradiation duration), a variety of paired one-on-one hierarchical plasmonic–photonic nanostructures were obtained, as summarized in Figure 1(c), for an Au nanocrown (AuNC) paired on a SiO_2_NS (AuNC@SiO_2_NS), a large Au nanosphere (AuNS) on a SiO_2_NS (AuNS@SiO_2_NS), and multiple small Au nanoparticles (AuNPs) distributed on a SiO_2_NS (AuNPs@SiO_2_NS).

**Figure 2:**
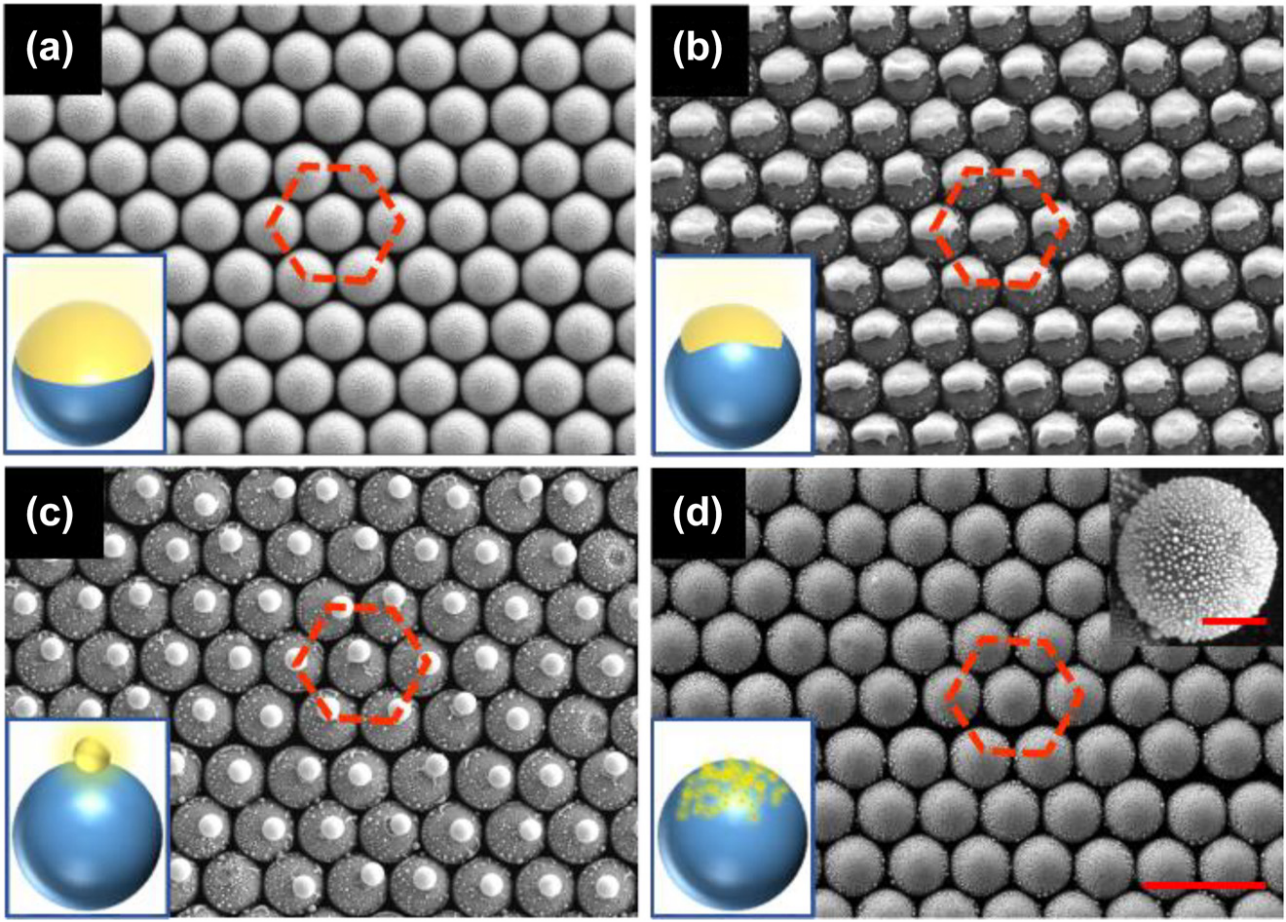
SEM images and schematical drawing of the arrays of (a) Au film (25 nm) coated SiO_2_NSs (500 nm), (b) AuNC@SiO_2_NS, (c) AuNS@SiO_2_NS, and (d) AuNPs@SiO_2_NS, prepared upon laser-induced dewetting with the same irradiation time of 700 ms under different laser fluence of 0, 10, 18, and 73 mJ/cm^2^, respectively. The inset in (d) is a magnified SEM image showing the morphology and distribution of small Au nanoparticles. The scale bars in (d) and the inset denote 1 μm and 200 nm, respectively.

The SEM images of the obtained hierarchical plasmonic–photonic nanostructure arrays are presented in Figure 2, upon laser-induced dewetting of 25 nm thick Au film on assembled monolayer SiO_2_NS array. Each SiO_2_NS is covered with an individual crescent (or hemispherical) Au film after deposition (the corresponding SEM images are shown in Figure S1). The vapor deposited Au films are typically with several to tens of nanometer roughness [16]. When exposed to the ns-pulsed laser, the Au film starts to dewet to form various Au nanostructures on SiO_2_NSs under different fluence. Figure 2(b)–(d) show the three typical Au nanostructures on the top of SiO_2_NS arrays constructed by the laser-induced dewetting. Under low laser fluence of 10–15 mJ/cm^2^, the one-on-one pairing of Au nanocrown on the top of a SiO_2_NS was formed [AuNC@SiO_2_NS, Figure 2(b)]. With the increase of laser fluence to the range of 17–30 mJ/cm^2^, a large Au nanosphere standing on each SiO_2_NS [AuNS@SiO_2_NS, Figure 2(c)] was obtained. Further increasing the laser fluence to 54–87 mJ/cm^2^, multiple small Au nanoparticles were observed on the top of one SiO_2_NS with relatively uniform size distribution [AuNPs@SiO_2_NS, Figure 2(d)].

The mechanism of the laser-induced dewetting driven *in situ* structural evolution from the Au film is similar to a thermal dewetting process, which can be well understood by the diffusion kinetics of the gold atoms over a dielectric metasurface or a rough substate [16]. The morphological evolution of Au nanostructures on the SiO_2_NSs is a function of the thermal accumulation determined by the laser fluence and irradiation duration. The minimum fluence required to induce the film melting depends on the thickness and roughness of the initial Au film. The hotspots created by these nanoripples as well as the curvature of SiO_2_NS help reduce the required minimum laser dewetting fluence. In our case, the closely packed SiO_2_NSs with EMDRs may also result in a significant thermal cumulative enhancement.

**Figure 3:**
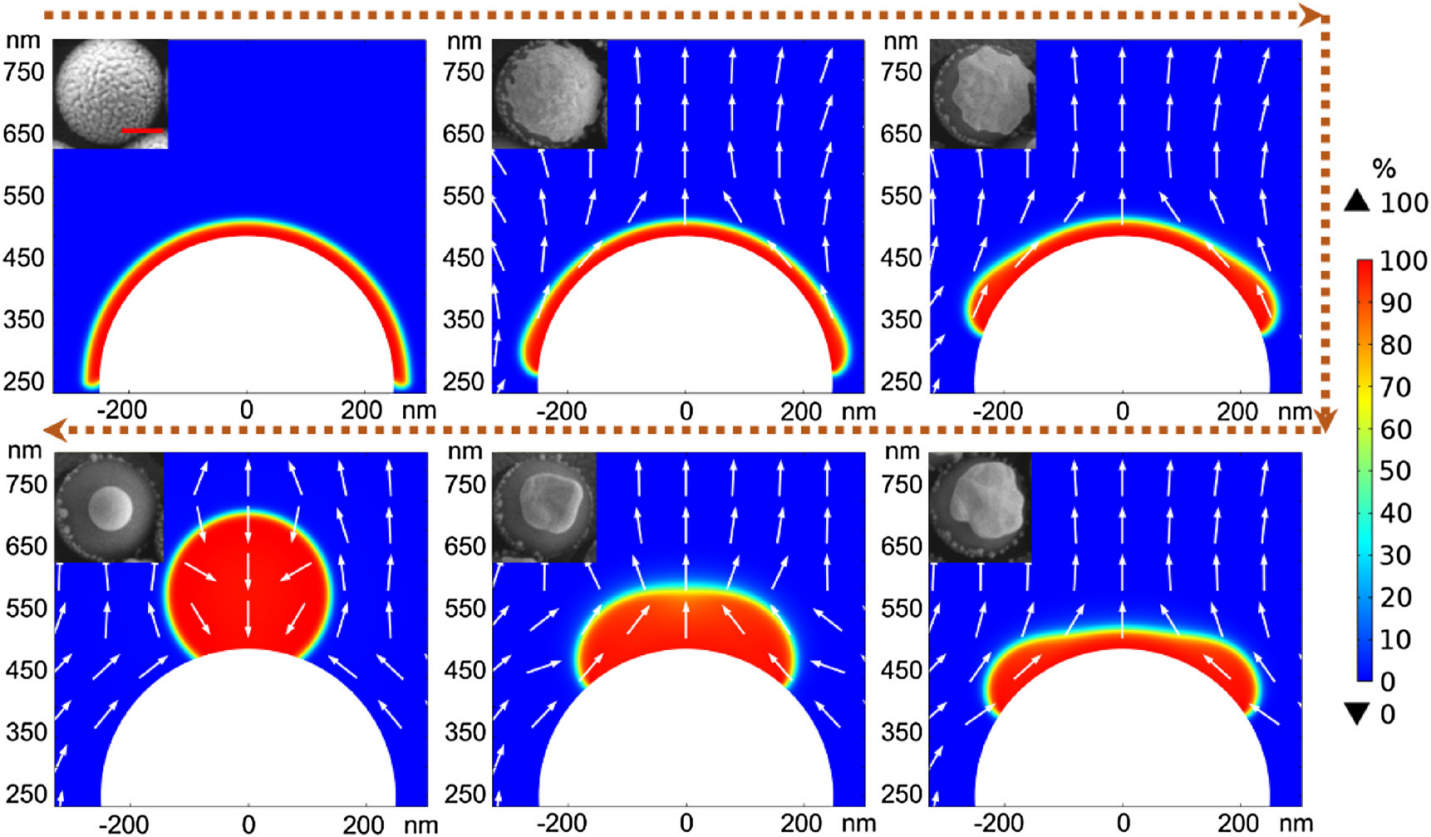
Simulated dynamic profiles of a dewetting process under the action of the surface tension of the molten Au film during the formation of AuNC@SiO_2_NS and AuNS@SiO_2_NS. The arrow direction corresponds to the flow field of the liquid gold atoms. The gold melting point (1064 °C) is chosen as the working temperature to ensure the liquid phase of the molten Au film in the simulation. Insets are the top-view SEM images of experimentally observed nanostructures during the dewetting process. The scale bar in inset represents 200 nm.

To clarify the mechanism of the laser-induced Au nanospheres formation, the Au nanostructure morphologies from a series of experiments were characterized using SEM, as shown in the insets of Figure 3. The samples obtained at different irradiation time in the range of 20 min to 2 h at a low laser fluence of 10 mJ/cm^2^ (melting threshold of the Au film) exhibit multiple intermediated states of the nanostructures. The low laser fluence induces slow thermal accumulation which allows us to observe the intermediated states of the nanostructure evolution as a function of irradiation time. To further figure out the intermediated state of the nanostructure, the flow field dynamics under the action of the surface tension of the molten Au film on a hemispherical curved substrate was modeled using a two-phase flow finite element analysis method (COMSOL Multiphysics software). The parameters used in the modeling include the Au density of 19.32 g/cm^3^, the molten Au film’s viscosity of 5.38 × 10^−3^ Pa/s, and the contact angle of molten Au on SiO_2_ surface of 120°. The ns-pulsed laser irradiation induced film dewetting is usually considered as a liquid-state dewetting, the morphology of Au film reshaping on the hemispherical SiO_2_NS surface can thus be summarized as the melting (phase transition from solid to liquid), the molten film (liquid phase) retraction, and the solidification (phase transition from liquid to solid) states.

Upon laser irradiation, the free electrons absorb the laser energy through inter- and intra-band transition and subsequently transfer heat to the lattices *via* electron–electron, electron–phonon, and phonon–phonon scattering. Because of the low thermal conductivity of air and SiO_2_NS, heat generated by laser is mostly confined in the Au films. When the laser energy raises the Au temperature beyond its melting point, the molten liquid Au film follows a classical spinodal dewetting behavior [31–33]. The combination of liquid surface tension and Laplace pressure [34] in curvature leads to an unstable fluctuation of liquid Au surface with a spinodal wave. Surface tension drives a liquid Au film to retract, and Laplace pressure induces the ruptures of a liquid Au film to form one or multiple islands.

At low laser fluence, the slight fluctuation cannot completely rupture a molten Au film; and thus, the whole piece of Au film retracts to form a large AuNS standing on the top of a SiO_2_NS. The isotropic curvature of monodisperse SiO_2_NS facilitates the one-on-one pairing to achieve uniform AuNS@SiO_2_NS array. In contrast, at high laser fluence, the amplitude of the surface fluctuation increases and finally reaches the critical characteristic length; therefore, the ripple Au film ruptures to multiple nano-islands which retract separately and form multiple small AuNPs sitting on one SiO_2_NS to achieve AuNPs@SiO_2_NS.

According to the relationship between the surface tension of Au [*γ*(Au)], and the temperature generated by laser irradiation (*T*) [35],

$$\gamma \left(Au\right)=1.15-0.14\times 10^{-3}\left(T-1064\right),$$

the surface tension decreases with the increase of temperature. As a result, the retraction speed of the molten Au nano-island slows down upon increasing laser fluence, which further facilitates the breakup of the liquid Au film to form multiple small AuNPs.

**Figure 4:**
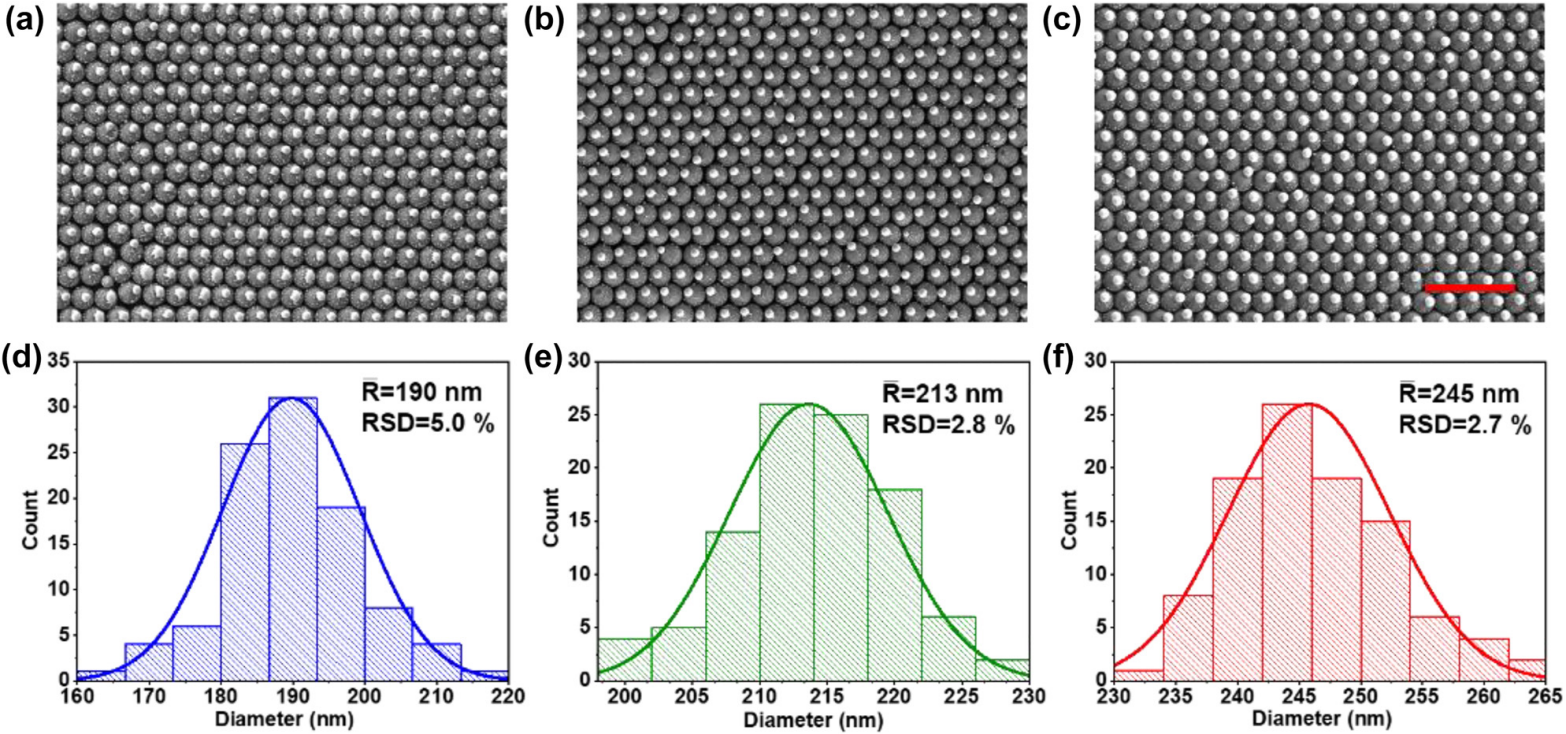
SEM images of the AuNS@SiO_2_NS arrays prepared by laser-induced dewetting of the Au films with thickness of (a) 20 nm, (b) 25 nm, and (c) 30 nm, upon 700 ms irradiation at different fluence of 15, 18, 21 mJ/cm^2^, respectively. (d–f) Corresponding size distribution diagrams of the obtained AuNSs. The scale bar represents 2 μm.

As shown in Figure 4, the formed AuNS size can be tuned by varying the deposited Au film thicknesses under an optimized laser-induced dewetting fluence. With the increase of Au film thickness, the formed AuNS diameter increases following a proportional relationship based on the mass conservation. The prepared AuNSs not only have excellent sphericity with a relatively uniform size distribution but also hold a well-ordered one-on-one pairing on SiO_2_NSs. This validates the possibility of precise control over the formed AuNS size by this proposed laser-induced dewetting method. In addition, as we know that the chemically synthesized AuNSs above 200 nm hardly process an even size distribution and perfect shape [36, 37]. This proposed method provides a rapid and simple method for controllable preparation of large size AuNSs with uniform size and spherical morphology.

**Figure 5:**
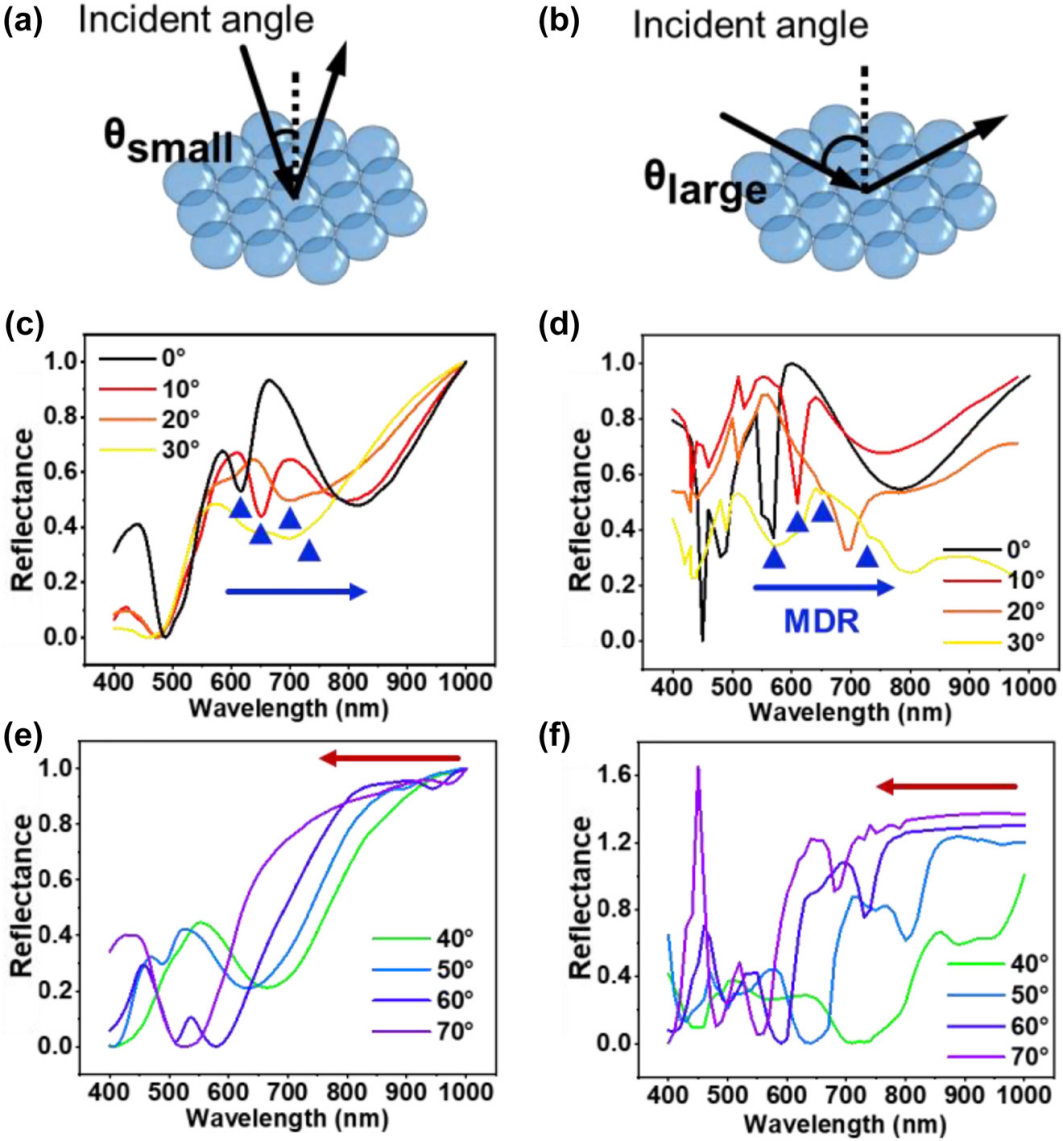
Schematic diagrams of two-dimensional photonic crystal array at (a) small incident angles and (b) large incident angles. (c) Experimentally measured and (d) simulated reflectance spectra of the monolayer SiO_2_NSs metasurface affected by EDR and MDR at incident angle of 0°–30°. (e) Experimentally measured and (f) simulated reflectance spectra of the monolayer SiO_2_NSs metasurface affected by PBG at incident angle of 40°–70°.

The fabricated hierarchical nanostructure arrays hold the photonic properties from the bottom of closely packed SiO_2_NSs. The monolayer SiO_2_NS array on silicon substrate exhibits shining structural colors at different viewing angles, which can be attributed to the multiple optical effects, such as scattering and diffraction. As the silicon substrate is opaque, angle-dependent reflectance spectra along the ordinary reflection direction were measured in our experiments. Considering that different angles of incident light affect the wave vector in two-dimensional plane, we conducted the theoretical and experimental studies on the angle-dependent reflectance spectra of the SiO_2_NS array at small incident angle [*θ*_small_ = 0°–30°, Figure 5(a)] and large incident angle [*θ*_large_ = 40°–70°, Figure 5(b)], respectively. At the small incident angle, the experimentally measured angle-dependent reflectance spectra of the SiO_2_NS array present a broad reflection band with a red-shifted reflection dip in the visible light range. To clarify the origin of the corresponding reflection features in different incident angles, the reflectance spectra as well as the electromagnetic field distribution were investigated using FDTD simulation on the monolayer SiO_2_NS array on silicon substrate.

According to the simulated results, when the incidence light is along *z*-axis, SiO_2_NS array exhibits strong electric dipole response (EDR) and magnetic dipole response (MDR) occurs at 554 and 567 nm, respectively (Figures S2 and S3). Both electric and magnetic field are mainly localized inside the SiO_2_ nanosphere. The similar profiles between simulated and measurement reflectance spectra induced that these strong dipole resonances and the high-order quadrupole resonances induced absorption contributed to the observed reflection dips in visible range. Besides, when the incident angle increases from 0° to 30°, EDR is slightly blue-shifted to UV range, while the MDR is dramatically red-shifted to NIR range (Figure 5(c) and (d)). As the EDRs are asymmetric and much weaker than the zero-incident angle, the measured spectra do not show the obvious EDR mode (Figure S4). But the similar red-shift trend of MDR mode in both simulated and measured spectra also strongly support that these reflectance spectra are related to the dipole resonance mode. With increasing the incident angle, the horizontal direction of the wave vector (in-plane) becomes dominated. Bragg diffraction of the in-plane periodic structures is mainly responsible for the overall reflectance spectra, which shows blue-shift of a broad reflection band with the increase of incident angle in Figure 5(e). The measured spectra show similar trend to the FDTD results in most part, and the large deviation between the calculated and measured reflectance spectra at incidence angle of 30–40° is ascribed to the absolute low reflectivity and weak dipole resonance. The reflection from the substate (silicon wafer in our experiment) cannot be subtracted completely and might influence on the measured results when the reflection is extremely low.

**Figure 6:**
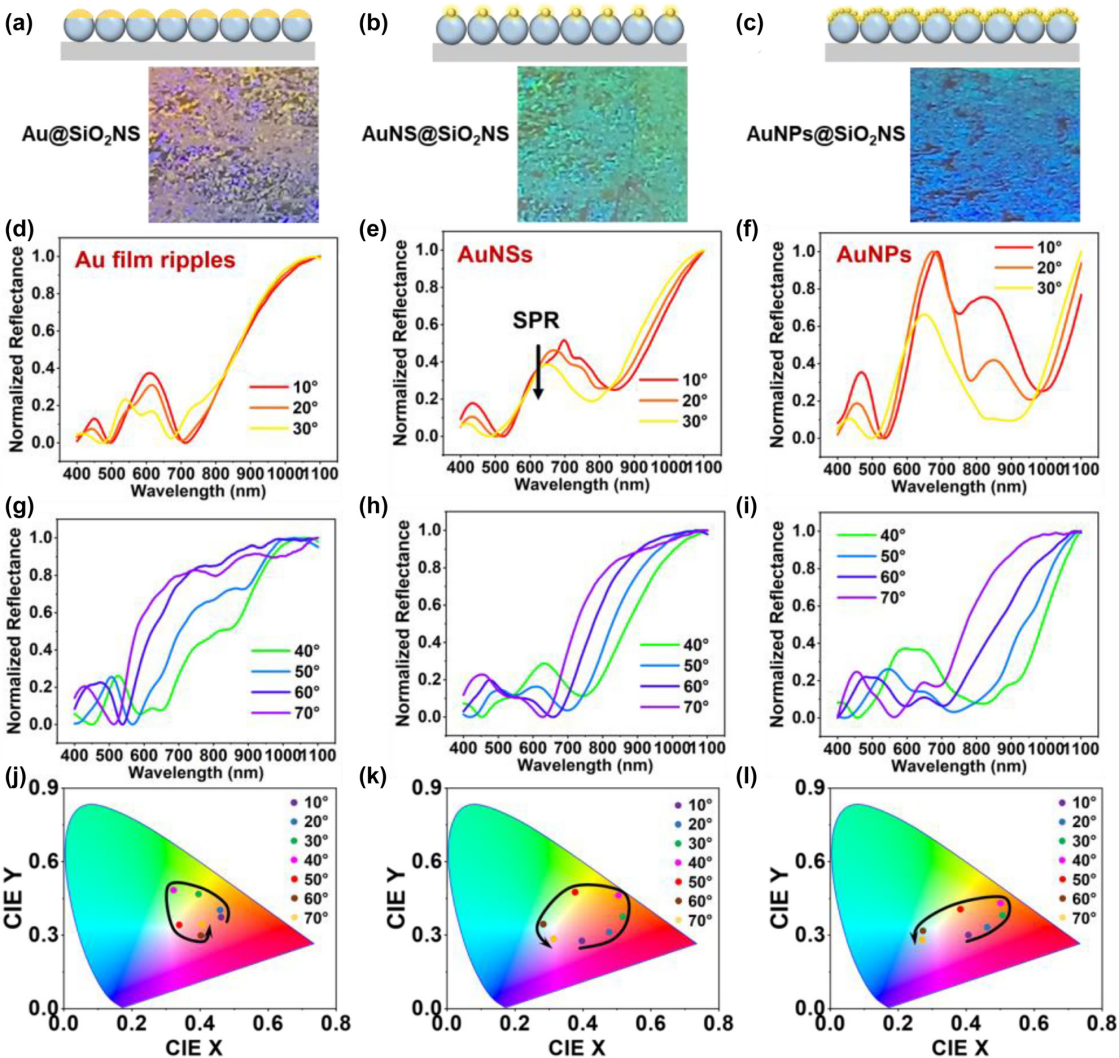
Schematic diagrams of the angle-dependent reflectance spectra measurement and optical images of monolayer (a) Au@SiO_2_NS, (b) AuNS@SiO_2_NS, and (c) AuNPs@SiO_2_NS arrays on silicon substrate at the same viewing angle. Corresponding reflectance spectra upon (d–f) small incident/reflection angles of 10°–30° and (g–i) large incident/reflection angles of 40°–70° (j–l) corresponding CIE chromaticity diagram obtained from (d–i).

Compared to the SiO_2_NS array, the Au@SiO_2_NS, AuNS@SiO_2_NS, and AuNPs@SiO_2_NS arrays demonstrate obvious synergistic effects of both photonic effects from the monolayer SiO_2_NS array and the SPR effect from the top Au nanostructures. The SPR spectra are strongly related to the shape and size of the Au nanostructures as well as the interparticle distance. The well-ordered one-on-one pairing of Au nanostructures on SiO_2_NSs facilitates the strong plasma resonance to tailor the reflectance spectra of the monolayer SiO_2_NS array. Figure 6(a)–(c) illustrate the visibly vivid colors exhibited by Au@SiO_2_NS, AuNS@SiO_2_NS, and AuNPs@SiO_2_NS arrays, under the same natural light. The last two samples were prepared by laser-induced dewetting of the same sample of 25 nm Au film covered 500 nm SiO_2_NS at different laser fluence. This indicates that the size and morphology of the obtained Au nanostructures dramatically influence on their optical properties. Figure 6(d)–(l) show the corresponding angle-dependent reflectance spectra, CIE chromaticity diagrams of the samples with the incident/reflected angle changing from 10° to 70° at a step of 10°.

As shown in Figure 6(d) and (g), the much lower reflection valley at around 700 nm in the reflection spectra of the Au@SiO_2_NS array is attributed to the plasma enhanced absorption according to the nanoripples of the Au film. As the Au nanostructures paired on the top of SiO_2_NSs, the plasmonic effect plays a much significant role in the reflection spectra at the small incident angles compared to those at the large incident angles. The similar SPR effect is also observed on the reflection spectra of AuNS@SiO_2_NS array, as shown in Figure 6(e) and (h). The large 213 nm AuNSs exhibit a much broad SPR band around 640 nm as indicated in the extinction spectra of AuNS in Figure S5. The reflection of SiO_2_NS array is attenuated by the SPR band of AuNSs due to the plasmonic induced absorption and scattering. The same effect is also observed on the AuNPs@SiO_2_NS array in Figure 6(f) and (i). In this case, the small multiple AuNPs (∼15 nm) exhibit a strong plasma induced scattering and absorption in visible-NIR range due to the hotspots created by the nanogaps of AuNPs. Due to the relatively uneven distribution of AuNPs on SiO_2_NSs, the plasma bands shift at different incident angles, leading to the significant variation of the absorption valleys observed on the AuNPs@SiO_2_NS array. Owing to the plasmonic-dielectric coupling between Au nanostructures and SiO_2_NS array, a rich range of color variation and significant color saturation are achieved in the hierarchical Au@SiO_2_NS, AuNS@SiO_2_NS, and AuNPs@SiO_2_NS arrays, as presented in the CIE coordinate diagram in Figure 6(j)–(l).

**Figure 7:**
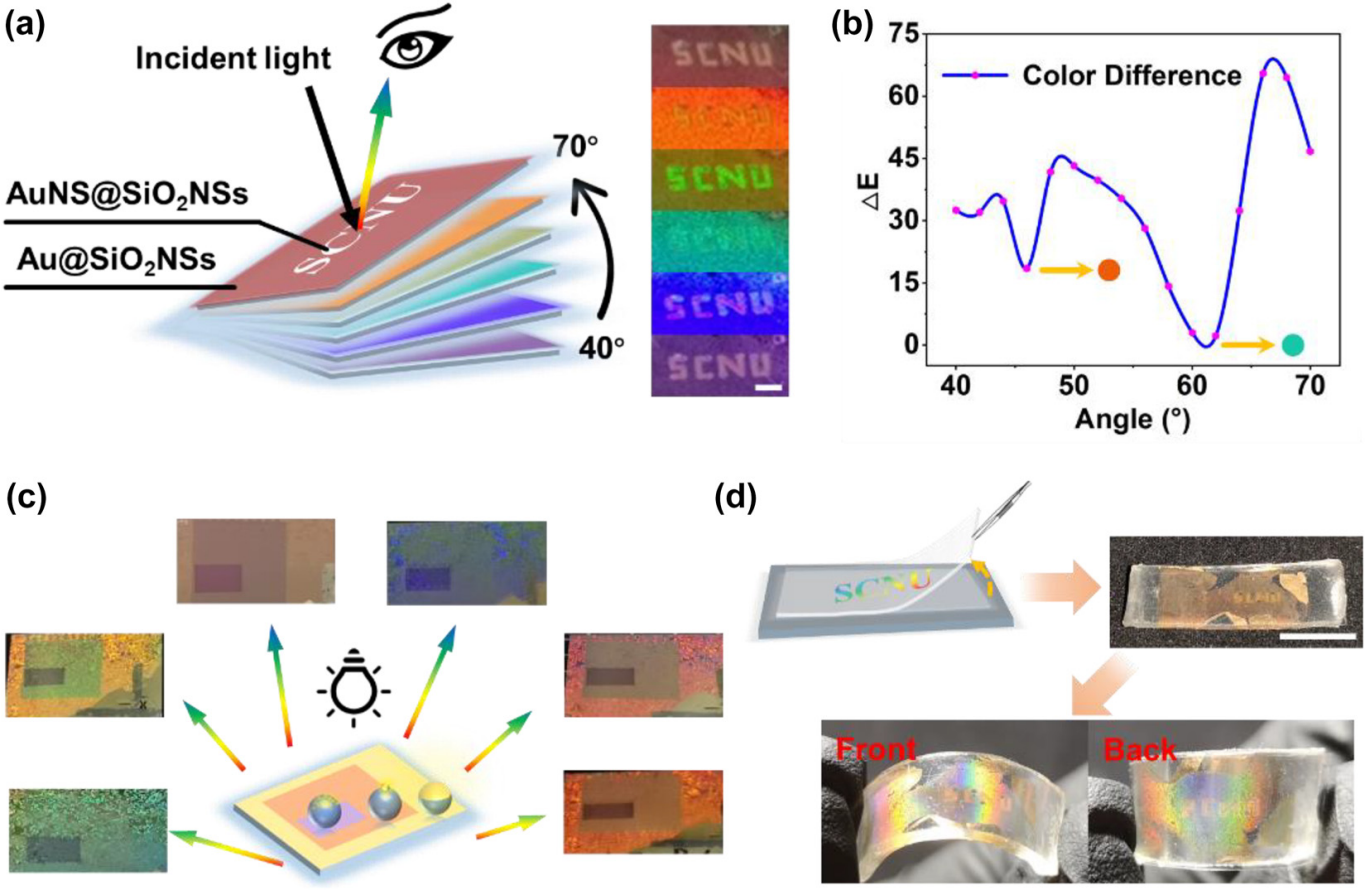
(a) Demonstration of anti-counterfeiting application of the angle-dependent optical differences of the two types of hierarchical nanostructures with the “SCNU” pattern of AuNS@SiO_2_NS array encoded in the substrate of Au@SiO_2_NS array. The scale bar denotes 2 mm. (b) Corresponding chromatic aberration upon viewing angle. (c) Schematic and optical images of the anti-counterfeiting films with the inner square of Au@SiO_2_NS array, the middle square of AuNS@SiO_2_NS array, and the outer square of AuNPs@SiO_2_NS array on a silicon substrate. (d) Transferring process and obtained hierarchical nanostructure arrays on a flexible substrate. The scale bar denotes 1 cm.

As mentioned above, the laser-induced dewetting combined with the 2D displacement platform or galvo mirrors allows us to directly “write” different hierarchical nanostructures with a well-defined micropattern in time or special sequence. As shown in Figure 7, the anti-counterfeiting application has been demonstrated by patterning a mixed hierarchical metasurface. The “SCNU” pattern of AuNS@SiO_2_NS array was “written” on the Au@SiO_2_NS array by using a laser beam, as presented in Figure 7(a). The patterned film showed diverse structural colors of both nanostructures. When rotating the metasurface within a small angle from 40° to 70°, the contrast of the “SCNU” pattern varied continuously and became indistinguishable for twice by exhibiting similar colors from the AuNS@SiO_2_NS and the Au@SiO_2_NS arrays. This can be interpreted by the two valleys (orange and cyan) exhibited in Figure 7(b), according to the calculated color difference Δ*E* in the optical photos when rotating the sample like that shown [38]. The invisible difference between the encoded pattern structure and encoding substrate structure can be achieved twice within a slight rotation angle, providing a great advanced strategy for information security for the anti-counterfeiting application.

A more complex metasurface with three types of hierarchical nanostructures including Au@SiO_2_NS, AuNS@SiO_2_NS, and AuNPs@SiO_2_NS arrays were also fabricated by simply varying the irradiation laser fluence in different regions, as demonstrated in Figure 7(c). Under natural light, the two square patterns of AuNS@SiO_2_NS and AuNPs@SiO_2_NS arrays encoded in an Au@SiO_2_NS array exhibit different colors with obvious color contrast. The color of the AuNS@SiO_2_NS array can also be hidden in the Au@SiO_2_NS array at certain viewing angles. This offers more possibility of increasing the capacity of information storage and the dimension of encryption in various fields.

In addition, thanks to the quick pulse laser-induced dewetting process without obvious heating effect on the surrounding areas, the constructed nanostructures can be directly “written” or easily transferred *via* a simple “pasting” and “peeling” process, as depicted in Figure 7(d). The well-patterned film with mixed nanostructure arrays was transferred to a transparent Acrylic Tape with strong adhesion on a flexible supporting substrate. The laser “written” pattern has been well reserved and observed on double sides. These results greatly expand the potentials of such hierarchical nanostructures and their mixtures on both rigid and flexible substrates.

## 4 Conclusions

In this work, various and patternable one-on-one pairing plasmonic–photonic hierarchical nanostructure arrays have been constructed using a facile and efficient method. The overall process involves colloidal assembly, film deposition, and laser irradiation, being available to either fabricate in a large area or “write” in a focused area with high controllability and stable reproducibility. The *in situ* structural evolution of the metal film dewetting is related to the transient thermal accumulation determined by the laser fluence and irradiation duration. The hotspots from nanoripples of Au film as well as the EMDRs of photonic crystal template significantly enhanced the thermal cumulative and thus decreased the required minimum laser dewetting fluence. The final appearance of Au nanostructures on SiO_2_NS array ranging from Au nanocrown to a large AuNS and multiple small AuNPs are determined by the surface tension and Laplace pressure of liquid molten Au film on the top of SiO_2_NS, following a spinodal dewetting behavior. The optical properties of the constructed hierarchical plasmonic–photonic nanostructure arrays demonstrated obvious synergistic effect of both photonic effects (EMDR and PBG) from the bottom monolayer SiO_2_NS array and SPR from the top Au nanostructures. Due to the plasmonic, the photonic, and the plasmonic–photonic coupling effects, the patterned hierarchical arrays created by different laser irradiation conditions exhibited unique pattern-dependent structural color variation. The anti-counterfeiting applications have been demonstrated by patterning one or more pairing nanostructures on the base nanostructure array. In addition, the hierarchical structure arrays and corresponding patterns could also be transferred onto different substrates, expanding its potentials for applying in flexible and transparent devices. This proposed method is simple, fast, and effective, and the achievable hierarchical structures and patterns are controllable and versatile, offering great potential for advanced optical multiplexing technologies.

## Supplementary Material

Supplementary Material Details
