# Supplementary material for: Programmable hierarchical plasmonic–photonic arrays via laser-induced film dewetting
Source: Nanophotonics. 2022 Jul 15;11(16):3641–51. doi: 10.1515/nanoph-2022-0272 (PMC11501294; doi:10.1515/nanoph-2022-0272)
Supplement: Supplementary file 1 — Supplementary Material Details [file j_nanoph-2022-0272_suppl.doc]

Zeyu Zheng, Yu Miao, Jiyuan Yao, Jiamei Chen, Jialin Wen, Xiaodan Chen, Yanxin Lu, Xiaofang Jiang*, Lingling Shui*

Programmable hierarchical plasmonic-photonic arrays *via* laser-induced film dewetting

**SUPPLEMENTARY FIGURES**

***Corresponding authors:** Lingling Shui, Guangdong Provincial Key Laboratory of Nanophotonic Functional Materials and Devices, School of Information and Optoelectronic Science and Engineering, South China Normal University, Guangzhou 510006, P. R. China, E-mail: [shuill@m.scnu.edu.cn](mailto:shuill@m.scnu.edu.cn); and Xiaofang Jiang, Laboratory of Quantum Engineering and Quantum Material, School of Physics and Telecommunication Engineering, South China Normal University, Guangzhou 510006, China, E-mail: [jiangxf@scnu.edu.cn](mailto:jiangxf@scnu.edu.cn).

**Zeyu Zheng, Jiyuan Yao, Jiamei Chen, Jialin Wen and Xiaodan Chen:** Guangdong Provincial Key Laboratory of Nanophotonic Functional Materials and Devices, School of Information and Optoelectronic Science and Engineering, South China Normal University, Guangzhou 510006, P. R. China.

**Yu Miao and Yanxin Lu:** Laboratory of Quantum Engineering and Quantum Material, School of Physics and Telecommunication Engineering, South China Normal University, Guangzhou 510006, China.

**Figure S1:** SEM images showing the **(a)** top-view and (b) side-view the of Au films (thickness = 25 nm) coated SiO2NS (D = 500 nm) array after thermal evaporation. The scale bars denote 1 μm and 500 nm, respectively.


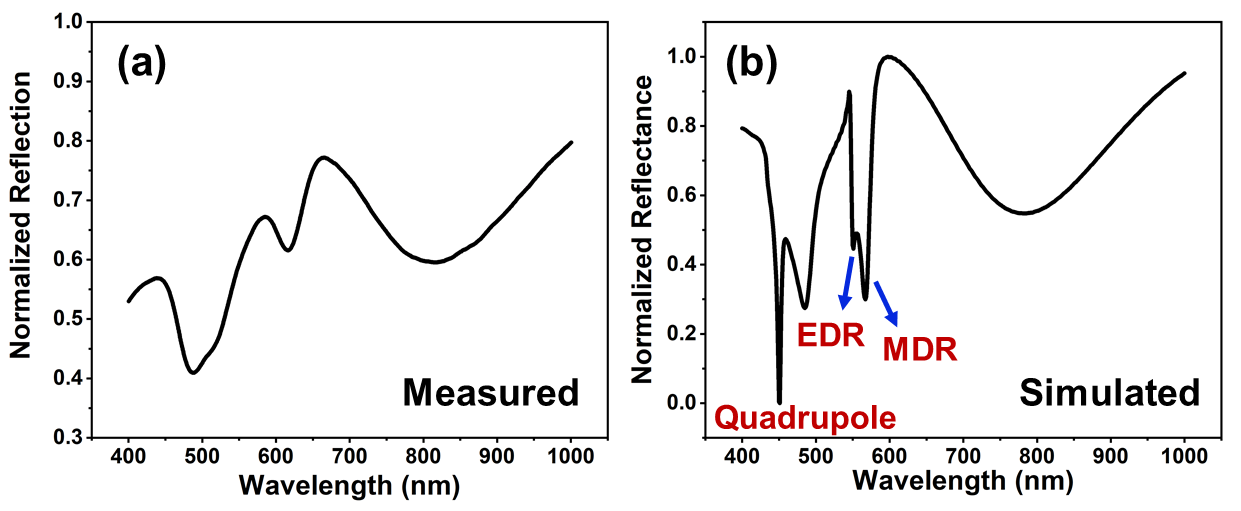


**Figure S2: (a)** Experimentally measured and **(b)** simulated reflectance spectra of the monolayer SiO_2_NSs metasurface affected by EDR and MDR at zero incidence angle.


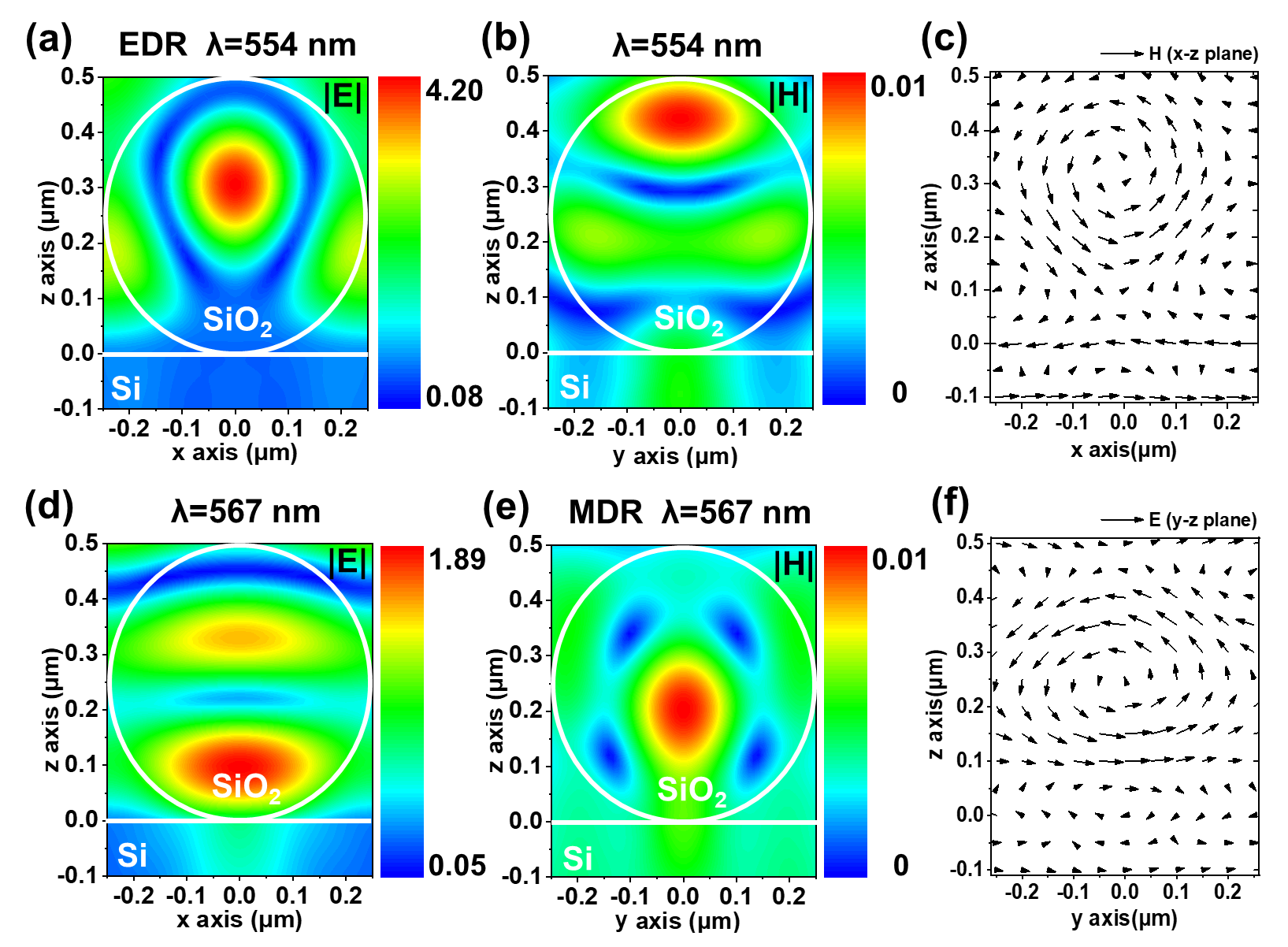


**Figure S3:** **(a)** Electric field distribution of the monolayer SiO_2_NS arrays in xz-plane at 554 nm. **(b)** Magnetic field distribution of the monolayer SiO_2_NS arrays in yz-plane at 554 nm. **(c)** Corresponding magnetic vector distribution in xz-plane. **(d)** Electric field distribution of the monolayer SiO_2_NS arrays in xz-plane 567 nm. **(e)** Magnetic field distribution of the monolayer SiO_2_NS arrays in yz-plane at 567 nm. **(f)** Corresponding electric vector distribution in yz-plane.


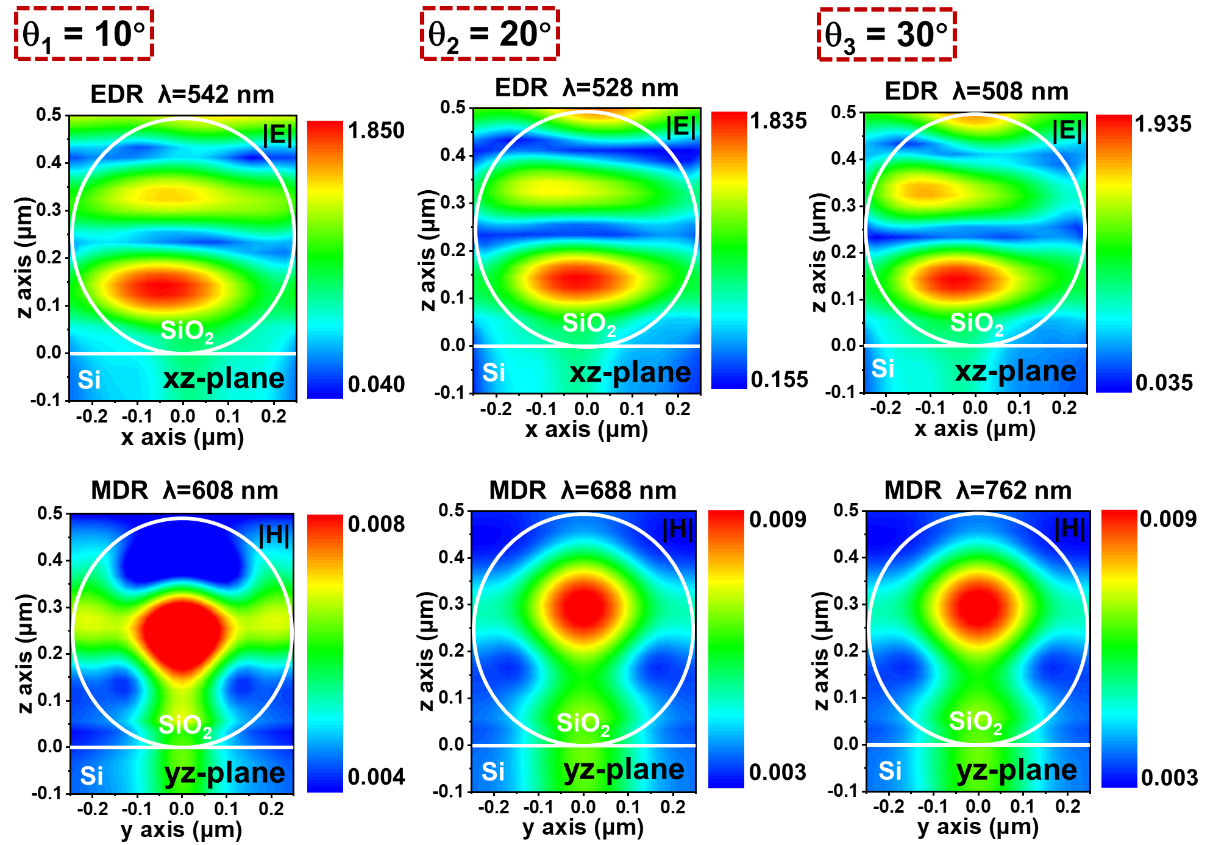


**Figure S4:** Electric and magnetic field distribution of the EDR and MDR at incidence angles of 10, 20 and 30°.


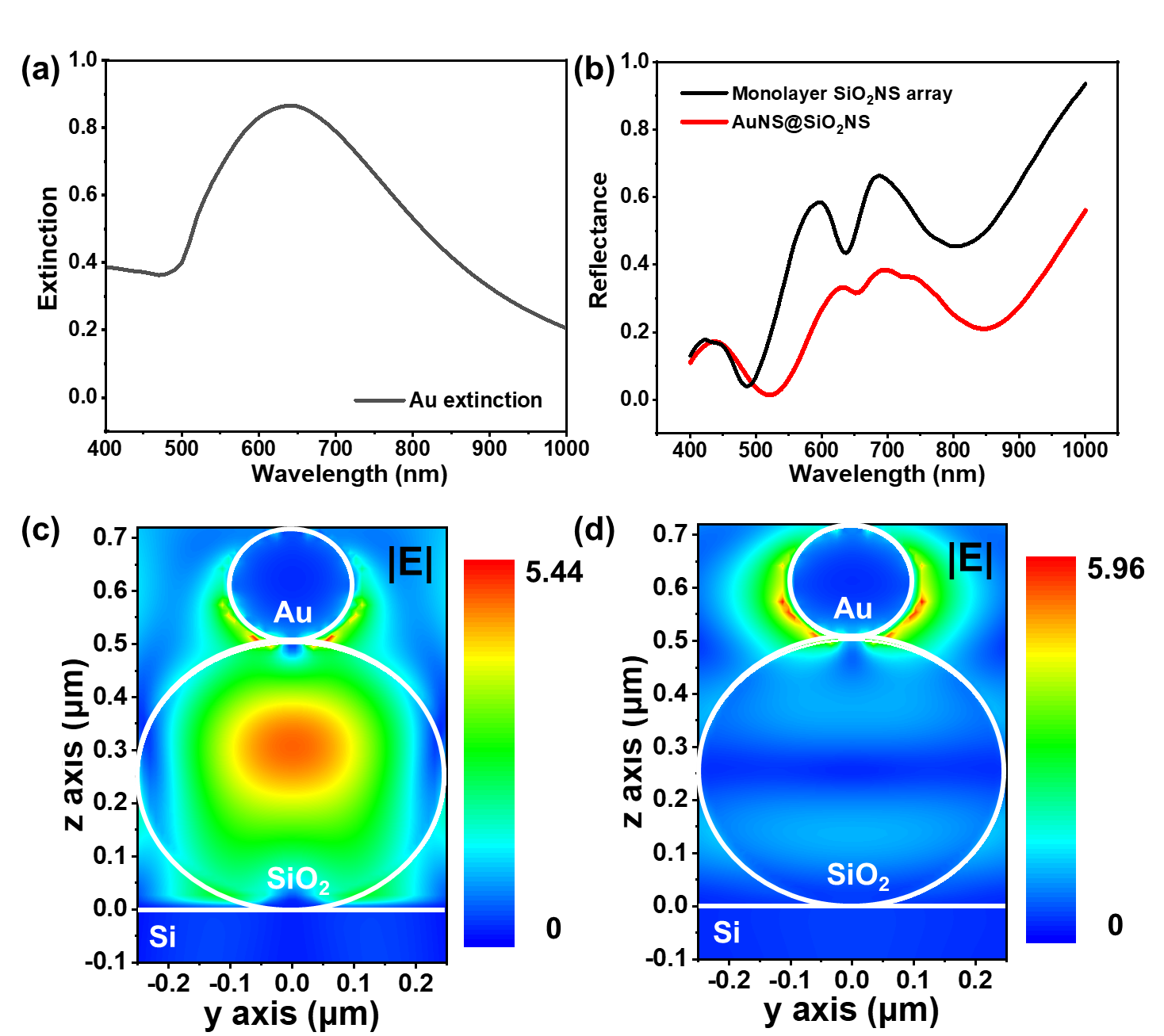


**Figure S5: (a)** Extinction spectrum of an AuNS (d = 213 nm). **(b)** Reflection spectra comparison between the monolayer SiO_2_NS array and the AuNS@SiO_2_NS hierarchical array under the near zero-incidence angle. Simulated electric filed distributions in the AuNS@SiO_2_NS nanostructure at **(c)** 550 nm and **(d)** 640 nm.


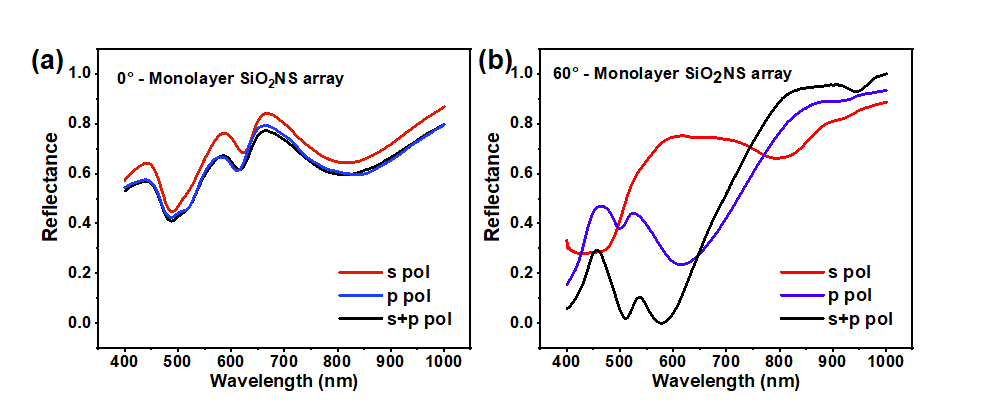


**Figure S6:** S/P-polarization dependent reflectance spectra of the monolayer SiO_2_NSs metasurface upon incident angles of **(a)** 0°and **(b)** 60°.


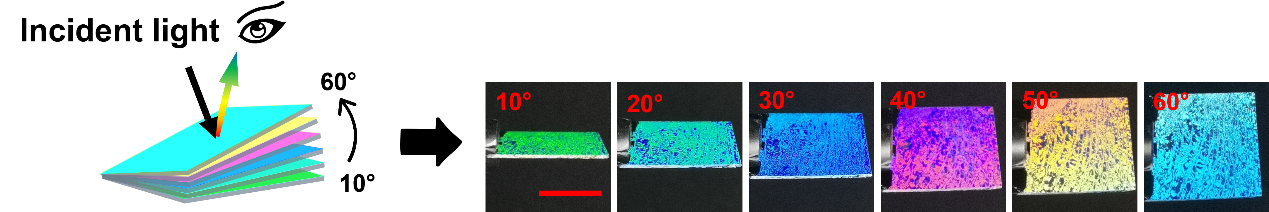


**Figure S7:** Schematic and optical images of the samples viewed at different angles by rotating the same sample from 10° to 60° with a step of 10° at a fixed viewing angle. The scale bar denotes 1 cm.


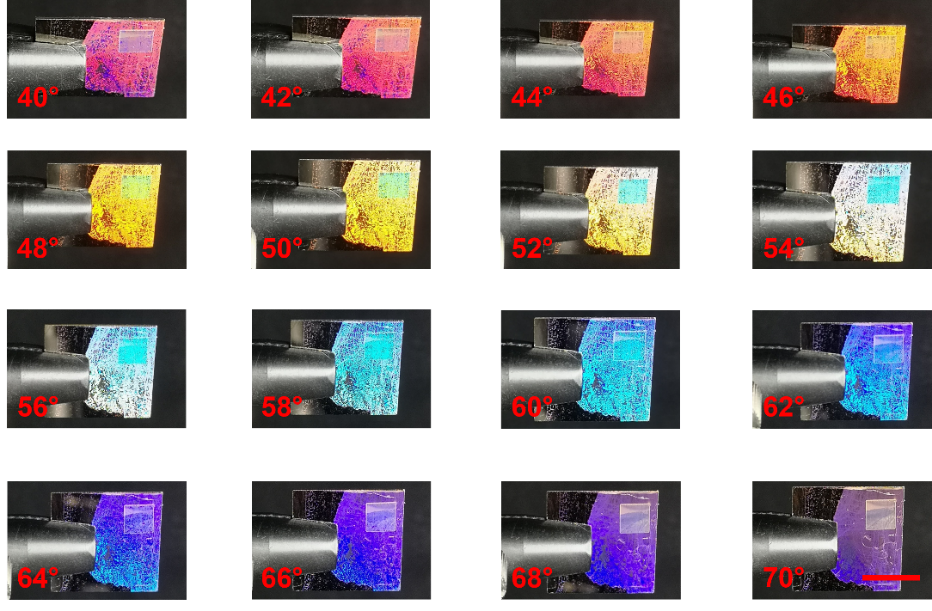


**Figure S8:** Photos of the samples with different scattering colors by rotating the sample from 40° to 70°. The sample is composed of AuNS@SiO_2_NS array on Si substrate. The scale bar denotes 1 cm.

**Table S1.** Parameters of Au and SiO_2_NS used in simulation

| Parameter | Numerical Value |
| --- | --- |
| Diameter (SiO_2_NS) | 500 nm |
| Density (SiO_2_NS) | 2.0 g/cm^3^ |
| Film Thickness (Au) | 25 nm |
| Density (Au) | 19.32 g/cm^3^ |
| Melting point (Au) | 1064 ℃ |
| Viscosity (molten Au) | 5.38×10^-3^ Pa/s |
| Contact angle (AuNS@SiO_2_NS) | 120° |
